# Ebola Virus Persistence Beyond Acute Infection: Could HIV-Associated Immune Dysfunction Influence Survivor Biology?

**DOI:** 10.3390/v18070755

**Published:** 2026-07-09

**Authors:** Francesco De Maria, Paolo Fusco, Alessandro Russo

**Affiliations:** 1 Infectious and Tropical Diseases Unit, Department of Medical and Surgical Sciences, “Magna Graecia” University of Catanzaro, 88100 Catanzaro, Italy; francescodemaria16@gmail.com; 2 Infectious and Tropical Diseases Unit, “Renato Dulbecco” Teaching Hospital of Catanzaro, 88100 Catanzaro, Italy

**Keywords:** Ebola virus persistence, Ebola virus disease survivors, HIV-associated immune dysfunction, immune-privileged reservoirs, viral persistence, post-acute Ebola syndrome

## Abstract

Ebola virus disease (EVD) has traditionally been considered an acute infection characterized by high mortality and severe systemic inflammation. However, growing evidence accumulated over the last decade has progressively challenged this view, demonstrating that Ebola virus may persist long after apparent clinical recovery within immune-privileged anatomical compartments, including the male genital tract, ocular tissues, central nervous system, and breast milk. Persistent viral reservoirs have been associated with prolonged RNA shedding, sexual transmission, recrudescence phenomena, and outbreak resurgence, highlighting the clinical and public health relevance of post-acute Ebola persistence. At the same time, increasing evidence suggests that EVD survivors frequently exhibit chronic inflammatory activation and long-lasting immune dysfunction. Persistent alterations involving cytokine signaling, T-cell responses, and antiviral immune regulation may contribute to incomplete viral clearance and reservoir maintenance. In this context, the potential interaction between Ebola virus persistence and HIV-associated immune dysregulation remains poorly explored despite the substantial geographical overlap between both infections in sub-Saharan Africa. This narrative review examines current evidence regarding Ebola virus persistence, immune-privileged reservoirs, survivor immune dysfunction, and persistence-associated transmission. Additionally, we discuss the biological plausibility that chronic immune activation, T-cell exhaustion, and impaired antiviral surveillance observed in people living with HIV (PWH) could theoretically influence persistence dynamics and long-term reservoir biology. Understanding these interactions may have implications for survivor monitoring, outbreak preparedness, and future research on post-acute viral reservoir diseases. Importantly, this review does not argue that HIV has been clinically established as a modifier of Ebola virus persistence. Rather, it examines Ebola virus persistence as an established post-acute phenomenon and considers whether HIV-associated immune dysregulation in people living with HIV (PWH) may represent a biologically plausible, but still untested, determinant of viral clearance and reservoir biology.

## 1. Introduction

Ebola virus disease (EVD) remains one of the most severe viral infections affecting humans, characterized by high mortality, systemic inflammation, endothelial dysfunction, and multiorgan involvement [1,2,3]. Historically considered an acute and self-limited infection among survivors, increasing evidence accumulated over the last decade has progressively challenged this paradigm. Viral persistence in immune-privileged compartments, prolonged inflammatory abnormalities, and delayed recrudescence events have reshaped the understanding of Ebola virus biology beyond the acute phase of disease [4,5,6].

The large West African outbreak of 2013–2016 represented a turning point in this regard, not only because of its unprecedented scale, but also because it generated the largest cohort of EVD survivors ever studied [7]. Follow-up investigations demonstrated that Ebola virus RNA may persist for months or even years after clinical recovery, particularly within the male genital tract, ocular tissues, central nervous system, and, more recently discovered, breast milk [5,8,9,10,11]. Importantly, viral persistence has not remained a purely virological observation. Molecular evidence supporting sexual transmission from survivors, together with outbreak flare-ups genetically linked to persistent viral reservoirs, has established persistence as a clinically and epidemiologically relevant phenomenon [12,13].

Among all documented reservoirs, the male reproductive tract represents the best characterized site of persistence. Data from several cohort studies conducted in Sierra Leone, Liberia, and Guinea demonstrated prolonged Ebola virus RNA detection in semen, occasionally extending well beyond the acute phase of disease [10,11,14,15]. These findings led to the implementation of national semen testing and counseling programs aimed at reducing secondary transmission risk among survivors [16]. The recognition that apparently recovered individuals could contribute to delayed transmission events profoundly influenced public health strategies and outbreak preparedness frameworks [12,16].

At the same time, growing evidence suggests that EVD survivors may experience persistent immune dysregulation long after viral clearance from blood compartments. Chronic inflammatory activation, altered cytokine profiles, and prolonged immune dysfunction have all been described in post-Ebola cohorts [17,18]. Such findings have fueled increasing interest in the mechanisms regulating incomplete viral clearance and long-term persistence within immune-privileged tissues.

In this evolving landscape, the potential interaction between Ebola virus persistence and HIV-associated immune dysfunction remains remarkably understudied. This gap is particularly relevant considering the geographical overlap between Ebola outbreaks and regions with high HIV prevalence in sub-Saharan Africa [19,20,21]. Although direct evidence remains limited, chronic immune activation, T-cell exhaustion, impaired cytotoxic responses, and persistent inflammatory signaling observed in people living with HIV (PWH) could theoretically influence the dynamics of Ebola virus persistence and reservoir maintenance [22,23,24,25,26,27].

It should be emphasized that this review does not imply that prolonged Ebola virus persistence has been definitively demonstrated in people living with HIV (PWH). Rather, it explores a biologically plausible but still under-investigated interaction between post-acute Ebola virus persistence and HIV-related immune dysregulation. In this context, evidence from other persistent viral infections in immunocompromised hosts may help frame how impaired immune surveillance could affect viral clearance and persistence dynamics [28,29].

**Figure 1:**
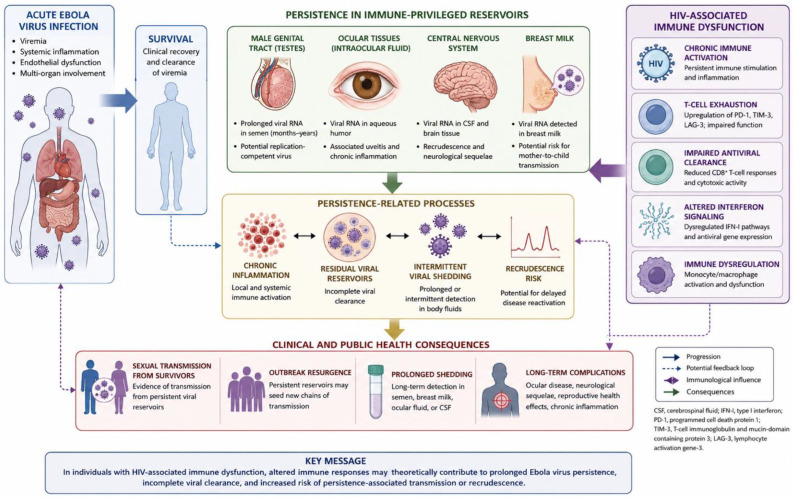
Proposed model of Ebola virus persistence and HIV-associated immune dysregulation. Conceptual overview illustrating the transition from acute Ebola virus disease to post-acute viral persistence within immune-privileged anatomical compartments, including the male genital tract, ocular tissues, central nervous system, and breast milk. Persistent viral reservoirs may contribute to chronic inflammation, intermittent viral shedding, and recrudescence phenomena. The figure additionally summarizes the potential role of HIV-associated immune dysfunction, including chronic immune activation, T-cell exhaustion, impaired antiviral clearance, altered interferon signaling, and immune dysregulation, as theoretical mechanisms potentially influencing Ebola virus persistence dynamics and long-term clinical consequences. Abbreviations: CNS, central nervous system; CSF, cerebrospinal fluid; IFN-I, type I interferon; PD-1, programmed cell death protein 1; TIM-3, T-cell immunoglobulin and mucin-domain containing protein 3; LAG-3, lymphocyte activation gene-3.

This narrative review examines current evidence regarding Ebola virus persistence in survivors, the role of immune-privileged reservoirs, the implications of prolonged viral shedding for transmission and outbreak resurgence, and the potential relevance of HIV-associated immune dysfunction in shaping persistence biology and long-term clinical outcomes. The proposed interaction between Ebola virus persistence, immune-privileged reservoirs, and HIV-associated immune dysregulation is illustrated in Figure 1. Given the heterogeneity of currently available data, the principal evidence domains supporting Ebola virus persistence and the proposed interaction with HIV-associated immune dysregulation are summarized in Table 1.

## 2. Biology of Ebola Virus Persistence

The recognition that Ebola virus may persist after apparent clinical recovery has fundamentally changed the traditional view of EVD as an exclusively acute infection. While viremia usually becomes undetectable in blood following recovery, increasing evidence indicates that the virus can remain within selected anatomical compartments for prolonged periods, particularly in immune-privileged tissues where local immune surveillance is reduced or highly regulated [4,5,36].

Immune-privileged sites such as the testes, eye, central nervous system, and placenta provide biological environments in which inflammatory responses are partially restricted in order to preserve organ function. Although physiologically beneficial, these characteristics may inadvertently facilitate incomplete viral clearance and prolonged persistence of viral material [4,5,37]. The male genital tract has emerged as the most extensively studied reservoir. Ebola virus RNA has repeatedly been detected in semen months after recovery, and several investigations have demonstrated persistence far beyond the acute phase of disease [8,10,11,14,15]. Experimental studies have additionally shown direct viral effects on testicular tissues and reproductive tract cellular responses, supporting the biological plausibility of local viral persistence [38,39].

Importantly, persistence does not necessarily imply the continuous presence of replication-competent virus. One of the major unresolved questions concerns the distinction between residual viral RNA fragments and biologically active virus capable of recrudescence or transmission. Most available studies rely on RT-PCR detection of viral RNA, an approach that is highly sensitive but does not distinguish between fragmented viral genomes and viable infectious virus. Consequently, prolonged RNA detection should not automatically be interpreted as evidence of ongoing viral replication or transmissibility. Demonstrating replication-competent virus requires complementary approaches, such as virus isolation, which remain technically challenging and have only rarely been performed in survivor cohorts. This methodological limitation represents one of the principal obstacles to accurately defining the true duration and clinical significance of Ebola virus persistence. Nevertheless, several observations suggest that persistence may occasionally retain clinical relevance. Molecular evidence of sexual transmission from survivors has confirmed that at least some persistent viral reservoirs remain infectious under specific circumstances [13]. Similarly, recrudescence events identified in survivors and animal models support the possibility that persistence may contribute to delayed disease reactivation [12,40].

Beyond the reproductive tract, ocular tissues have also received considerable attention. Ebola-associated uveitis and other ophthalmologic complications are now well-recognized sequelae among survivors [41,42,43]. Viral RNA persistence has been documented within intraocular fluids, and experimental models demonstrated associations between persistent intraocular viral material and severe inflammatory disease [44]. Mechanistic studies further suggest that Ebola virus may directly alter blood-retinal barrier integrity, potentially facilitating local viral maintenance and chronic inflammation [43]. These findings highlight the complex interaction between viral persistence and immune-mediated tissue injury.

Neurological persistence represents another emerging area of concern. Ebola virus neuroinvasion has been increasingly recognized both in human survivors and experimental models [40,45,46,47]. Viral persistence within central nervous system compartments may contribute to delayed neurological complications, encephalitic presentations, or recrudescence phenomena occurring after apparent systemic recovery [40,47]. Although the exact mechanisms remain incompletely understood, the CNS appears capable of serving as a long-term sanctuary site for viral persistence.

More recently, additional reservoirs have been identified outside the traditionally recognized immune-privileged organs. The detection of Ebola virus persistence in breast milk has raised concerns regarding maternal-infant transmission and highlighted the limited evidence currently available regarding breastfeeding management in survivors [9,48]. These observations underscore how persistence biology extends beyond isolated organ systems and may have broader reproductive and public health implications.

Importantly, persistence appears to be highly heterogeneous between individuals. Duration of viral RNA detection varies considerably across studies, and the determinants regulating prolonged shedding remain poorly understood [10,15]. Host immune status, severity of acute disease, tissue-specific factors, and underlying comorbidities may all influence persistence dynamics. Such variability has fueled growing interest in identifying conditions capable of impairing viral clearance or favoring reservoir maintenance.

Beyond tissue-specific immune privilege, the establishment and maintenance of Ebola virus persistence are also likely influenced by intracellular host–cell pathways regulating viral trafficking, antigen processing, lysosomal degradation, and innate immune sensing. Increasing evidence indicates that the endolysosomal system represents a critical checkpoint not only for Ebola virus entry but also for intracellular viral processing and the initiation of antiviral immune responses. Following internalization through macropinocytosis, Ebola virus undergoes sequential maturation within the endolysosomal compartment, where proteolytic processing of the viral glycoprotein enables its interaction with the cholesterol transporter Niemann-Pick C1 (NPC1), an essential intracellular receptor required for membrane fusion and productive infection [31].

Although NPC1 has been primarily investigated in the context of viral entry, the endolysosomal pathway may also influence subsequent events that determine whether viral material is efficiently degraded or retained within long-lived tissue reservoirs. Endosomal maturation, lysosomal function, vesicular trafficking, and antigen-processing pathways collectively regulate the balance between intracellular viral destruction and immune recognition. In addition, endolysosomal compartments serve as important platforms for innate immune sensing through endosomal pattern-recognition receptors, thereby linking intracellular viral processing with the activation of antiviral immune responses. Dysregulation of these mechanisms could therefore contribute to incomplete viral clearance, particularly within immune-privileged tissues where local inflammatory responses are physiologically constrained [31].

These observations add an important mechanistic dimension to the current understanding of Ebola virus persistence. Rather than acting solely as passive sites of immune escape, tissues such as the testes, eye, and central nervous system may provide intracellular environments in which viral processing, antigen presentation, and antiviral surveillance are less efficient, thereby facilitating prolonged maintenance of viral material. This intracellular perspective complements the anatomical concept of immune privilege by suggesting that long-term persistence is likely determined not only by the tissue microenvironment but also by host–cell mechanisms regulating viral processing and antiviral immunity. Overall, these findings indicate that Ebola virus persistence is shaped by the interplay between tissue-specific immune privilege and intracellular host–cell pathways, providing a mechanistic framework that links anatomical reservoirs with the intracellular fate of viral material.

The biological relevance of these pathways is further highlighted by experimental studies showing that pharmacological disruption of endolysosomal cholesterol trafficking using clinically approved agents such as itraconazole markedly impairs Ebola virus infection through interference with NPC1-dependent intracellular trafficking [30]. These findings illustrate that intracellular trafficking pathways are not only mechanistically important but may also represent therapeutically targetable host factors.

More broadly, growing interest in host-directed antiviral therapies has highlighted intracellular trafficking pathways as attractive therapeutic targets because they interfere with essential host mechanisms required for viral replication while potentially reducing the likelihood of antiviral resistance [49]. Although these strategies have primarily been investigated in the setting of acute infection, they further emphasize the importance of intracellular host–cell pathways in Ebola virus biology and suggest that they may also represent promising targets for future studies investigating the mechanisms governing viral persistence and long-term reservoir maintenance.

Collectively, current evidence suggests that Ebola virus persistence should no longer be considered an exceptional or incidental finding. Instead, it is increasingly recognized as a central component of post-acute Ebola biology, in which anatomical immune privilege, intracellular host–cell pathways, and host immune responses interact to determine long-term viral clearance and reservoir maintenance. However, important uncertainties remain regarding the duration, biological significance, and infectivity of persistent viral material, as much of the available evidence is based on molecular detection rather than direct demonstration of replication-competent virus. Addressing these knowledge gaps will require standardized longitudinal studies integrating virological, immunological, and clinical data.

**Figure 2:**
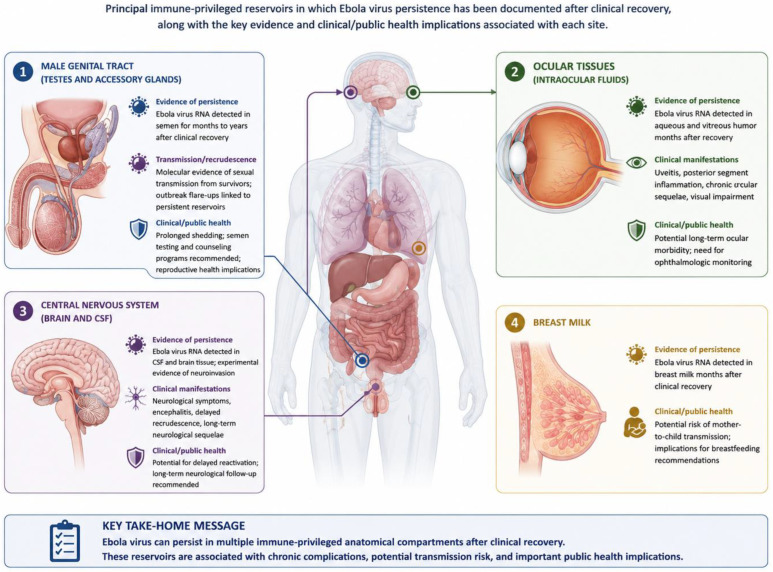
Immune-privileged anatomical sites involved in Ebola virus persistence. Schematic representation of the principal immune-privileged anatomical compartments in which Ebola virus persistence has been documented after clinical recovery. The figure highlights the male genital tract, ocular tissues, central nervous system, and breast milk as potential reservoir sites associated with prolonged viral RNA detection, chronic inflammatory complications, recrudescence phenomena, or persistence-associated transmission risk. Abbreviations: CNS, central nervous system; RNA, ribonucleic acid.

Taken together, these mechanisms have important implications for survivor medicine, outbreak preparedness, and the development of future host-directed therapeutic strategies aimed at improving viral clearance and limiting reservoir persistence. The principal immune-privileged anatomical sites involved in Ebola virus persistence are illustrated in Figure 2.

## 3. Sexual Transmission and Outbreak Resurgence

The possibility that Ebola virus persistence could contribute to delayed transmission events was initially considered largely theoretical. However, over the past decade, molecular and epidemiological evidence has increasingly shown that, under certain circumstances, survivors may remain a potential source of secondary transmission following apparent clinical recovery [4,5,32,50].

The strongest evidence supporting post-recovery transmission involves the male genital tract. Multiple studies identified prolonged Ebola virus RNA persistence in semen, occasionally extending for many months or even years after acute infection [10,11,14,15]. Methodological studies suggested that laboratory processing techniques, including semen sample pelleting, may improve the sensitivity of Ebola virus RNA detection in survivor cohorts [51]. Although RNA detection alone does not necessarily indicate infectivity, several investigations provided compelling evidence linking persistent viral reservoirs to documented sexual transmission events [13]. Genomic analyses comparing viral sequences from survivors and newly infected individuals demonstrated near-identical strains, strongly supporting direct transmission from persistent reservoirs rather than new zoonotic spillover events [12,13].

These findings substantially changed the understanding of Ebola outbreak dynamics. Traditionally, epidemic resurgence was primarily attributed to new animal-to-human transmission events. However, the 2021 Guinea outbreak challenged this assumption. Genomic investigations suggested that the outbreak likely originated from viral persistence in a survivor infected several years earlier rather than from a novel zoonotic introduction [12]. This observation introduced a new epidemiological paradigm in which persistent human reservoirs may occasionally contribute to outbreak re-emergence even after prolonged periods without active transmission. Key evidence supporting persistence-associated transmission and outbreak resurgence is summarized in Table 2.

The public health implications of such findings are considerable. Persistence-associated transmission complicates outbreak elimination strategies and extends the temporal horizon of surveillance efforts. Survivors may require prolonged follow-up, counseling, and testing programs aimed at minimizing secondary transmission risk while simultaneously avoiding stigma and social marginalization [47,53]. In response to these concerns, several national and international programs implemented semen testing initiatives for male survivors, particularly in West Africa following the 2013–2016 outbreak [16].

These programs marked an important step toward integrating survivor care with transmission prevention. Longitudinal semen-testing cohorts have contributed to delineating the duration of viral persistence, identifying behavioral risk factors, and refining counseling strategies on sexual health practices among survivors [10,16,50]. Importantly, these interventions also revealed substantial psychological and social challenges associated with prolonged persistence monitoring. Fear of transmission, social isolation, and uncertainty regarding reproductive health emerged as recurring concerns among survivors enrolled in follow-up programs [33,53].

Despite these advances, major uncertainties remain. The precise determinants regulating prolonged shedding are still incompletely understood, and the relationship between detectable RNA and true infectious potential remains variable across individuals and timepoints [2,34,35]. Furthermore, it is unclear whether host-specific factors, including underlying immune dysfunction, may influence persistence duration or transmissibility.

Experimental models have provided additional insight into the biological plausibility of persistence-associated transmission. Animal studies demonstrated prolonged viral presence within reproductive tissues and supported the role of the male genital tract as a potential long-term reservoir [38,39]. Similarly, investigations involving ocular and central nervous system compartments further reinforced the concept that immune-privileged sites may sustain persistent viral material under conditions of incomplete immune clearance [36,40,44]. It is important to emphasize that persistence-related transmission should not be interpreted as a common mechanism of Ebola spread.

Available evidence suggests that such events remain relatively uncommon compared with the intense transmission dynamics observed during acute outbreaks. Nevertheless, even rare persistence-associated transmission events may carry major epidemiological consequences due to the high pathogenicity of Ebola virus and the potential for renewed transmission chains [12,13].

In this context, Ebola persistence increasingly resembles a broader paradigm observed in other viral infections, in which residual reservoirs may contribute to prolonged shedding, delayed recrudescence, or intermittent transmission under conditions of incomplete immune control [28,29]. This conceptual framework becomes particularly relevant when considering populations characterized by chronic immune dysregulation, including people living with HIV.

The growing recognition that survivors may harbor persistent viral reservoirs long after clinical recovery has therefore transformed survivor medicine into an integral component of outbreak preparedness. Surveillance strategies can no longer focus exclusively on acute infection control but must also address the long-term biological and epidemiological consequences of viral persistence.

## 4. Persistent Immune Dysfunction After Ebola Virus Disease

Although Ebola virus disease has traditionally been viewed as an acute infection, growing evidence indicates that immunological alterations may persist long after apparent clinical recovery. In many survivors, recovery from viremia does not necessarily coincide with complete restoration of immune homeostasis. Instead, persistent inflammatory activation and long-term immune dysregulation appear to represent an important component of post-Ebola biology [6,17,18,33].

Several survivor cohorts have documented sustained abnormalities involving both innate and adaptive immune responses months or even years after acute infection [17,18]. Persistent elevation of inflammatory mediators, altered cytokine signaling, and immune activation profiles have been described in association with chronic symptoms and post-acute clinical sequelae [17,18]. Importantly, some studies reported associations between inflammatory signatures and the persistence of Ebola virus RNA within semen, suggesting a potential relationship between prolonged immune activation and incomplete viral clearance [17].

Among the most significant observations, Wiedemann and colleagues demonstrated evidence of severe and long-lasting immune dysfunction in Ebola survivors, characterized by persistent alterations in immune cell populations and inflammatory pathways long after recovery from acute disease [18]. These findings challenged the assumption that immune restoration necessarily follows viral clearance and instead suggested that Ebola infection may induce durable immunological remodeling.

The mechanisms responsible for persistent immune dysregulation remain incompletely understood. Several hypotheses have been proposed, including residual antigenic stimulation from persistent viral reservoirs, chronic endothelial activation, tissue-specific inflammatory responses, and dysregulated antiviral immunity [3,17,18]. Experimental studies also suggest that Ebola viral proteins may directly interfere with immune signaling pathways. For example, Ebola VP40-containing exosomes have been shown to induce immune cell dysfunction, potentially contributing to sustained inflammatory alterations even in the absence of active systemic infection [50].

Persistent immune abnormalities may also help explain the remarkable heterogeneity observed among survivors. While some individuals recover with minimal long-term consequences, others experience prolonged multisystem symptoms, inflammatory complications, or persistent viral shedding [6,17,33]. The coexistence of chronic inflammation and viral persistence raises the possibility that incomplete immune control may facilitate reservoir maintenance within immune-privileged tissues.

Notably, immune dysfunction after EVD does not appear to be restricted to a single organ system. Ocular complications, neurological sequelae, reproductive tract persistence, and systemic inflammatory manifestations collectively suggest a broader syndrome involving chronic immune perturbation and tissue-specific inflammatory injury [33,42,43,47]. This multisystem involvement further supports the concept that post-Ebola disease extends beyond isolated viral persistence and instead represents a complex interaction between residual viral reservoirs and host immune responses.

From a biological perspective, these findings create a particularly relevant framework for considering the potential impact of pre-existing immune dysregulation. Conditions characterized by chronic inflammation, impaired cellular immunity, or altered antiviral surveillance may theoretically influence the dynamics of Ebola persistence and clearance. In this regard, HIV-associated immune dysregulation offers a clinically relevant context for exploring these interactions.

Indeed, immune dysregulation in PWH may persist despite suppressive antiretroviral therapy, with features including persistent immune activation, T-cell exhaustion, altered cytokine signaling, and incomplete immune restoration [22,23,24,25,26,27]. Many of these mechanisms overlap conceptually with pathways implicated in post-Ebola immune dysfunction. Although direct evidence remains limited, such overlap raises important questions regarding whether HIV-associated immune dysregulation could modify the duration, compartmentalization, or clinical consequences of Ebola virus persistence.

Furthermore, lessons derived from other persistent viral infections in immunocompromised hosts provide additional biological plausibility for this hypothesis. Prolonged viral shedding, accelerated intrahost viral evolution, and delayed clearance have all been observed in chronically immunosuppressed individuals infected with a variety of RNA viruses [28,29]. While Ebola virus differs substantially from respiratory or chronic viral pathogens, these parallels reinforce the broader concept that impaired immune surveillance may alter persistence dynamics in selected hosts.

Taken together, current evidence increasingly supports the view that Ebola survivorship should not be interpreted solely through the lens of post-infectious sequelae. Instead, persistent immune dysfunction appears to represent a central biological feature of the post-acute phase of disease, potentially interacting with residual viral reservoirs in ways that remain only partially understood.

## 5. HIV-Associated Immune Dysfunction as a Potential Modifier of Ebola Persistence

The potential interaction between Ebola virus persistence and HIV-associated immune dysfunction remains poorly explored despite its possible clinical and epidemiological relevance. This gap is particularly notable considering the substantial geographical overlap between Ebola outbreaks and regions with high HIV prevalence in sub-Saharan Africa [19,20,21]. While direct evidence remains limited, several biological and immunological mechanisms support the plausibility that HIV-associated immune dysregulation in PWH could influence post-Ebola viral clearance and persistence dynamics. However, current knowledge is derived predominantly from indirect immunological observations rather than prospective clinical studies specifically evaluating Ebola survivors with HIV.

In PWH, persistent immune activation may continue despite suppressive antiretroviral therapy, representing a key feature of HIV-associated immune dysregulation [22,23,27]. Chronic inflammatory signaling, T-cell exhaustion, mitochondrial dysfunction, altered cytokine responses, and impaired antiviral immunity have all been extensively documented in PWH [22,23,24,25,26,27]. Importantly, many of these pathways overlap conceptually with the persistent immune abnormalities observed in Ebola survivors. The principal mechanisms of HIV-associated immune dysfunction potentially relevant to Ebola virus persistence are summarized in Table 3.

Under physiological conditions, viral clearance relies on the coordinated activity of innate and adaptive immune responses. Cytotoxic CD8^+^ T cells, macrophage activation, interferon signaling, and tissue-specific immune surveillance contribute to the elimination of infected cells and residual viral reservoirs. In PWH, persistent immune dysregulation and exhaustion phenotypes may interfere with these processes, potentially reducing the efficiency of viral clearance [23,24,25,26,27]. This could, in theory, favor the maintenance of Ebola virus within immune-privileged compartments.

The possibility that HIV-associated immune dysfunction may influence Ebola persistence is supported, albeit indirectly, by observations from other persistent viral infections. Immunocompromised hosts infected with RNA viruses have demonstrated prolonged shedding, delayed clearance, and enhanced intrahost viral evolution under conditions of impaired immune control [28,29]. Although Ebola virus differs biologically from respiratory viruses such as SARS-CoV-2, these findings reinforce a broader principle whereby defective antiviral immunity may alter persistence dynamics and reservoir stability.

To date, direct clinical evidence on the relationship between HIV-associated immune dysregulation and prolonged Ebola virus persistence remains very limited. To date, one of the most notable reports described Ebola virus RNA persistence in semen from an Ebola survivor with HIV, raising questions about whether underlying immune dysfunction may affect viral clearance [52]. Although a single observation cannot establish causality or demonstrate a consistent association, it underscores a clinically relevant but largely underexplored area that warrants further investigation through adequately powered prospective studies.

Beyond generalized immune activation, several HIV-associated mechanisms may be particularly relevant to Ebola persistence biology. Chronic interferon signaling has been shown to impair metabolic function and antiviral responses in CD8^+^ T cells during HIV infection [24]. Similarly, immune checkpoint activation and exhaustion pathways may contribute to incomplete elimination of infected cells and persistent reservoir maintenance [25,26]. Persistent inflammation and altered monocyte/macrophage activation additionally represent important components of HIV immunopathogenesis that could theoretically influence tissue-specific viral persistence [22,27,54].

The concept of viral reservoirs further strengthens this theoretical framework. HIV persistence itself is driven by long-lived cellular and tissue reservoirs capable of evading immune clearance despite prolonged therapy [55]. Although Ebola virus does not establish chronic infection in the same manner as HIV, parallels involving sanctuary sites, immune privilege, and incomplete viral eradication provide an interesting conceptual bridge between the two conditions. In both settings, tissue compartmentalization and local immune regulation appear to play central roles in persistence biology.

Another relevant consideration concerns the possibility that HIV-related immune dysfunction may not only affect persistence duration but also modulate the inflammatory consequences of residual viral reservoirs. Persistent immune activation following Ebola recovery has already been associated with chronic symptoms and systemic inflammatory alterations [17,18]. In PWH, pre-existing inflammatory dysregulation could theoretically amplify or prolong such post-acute immune disturbances.

At present, however, the available evidence remains insufficient to define whether HIV-associated immune dysregulation influences Ebola virus persistence outcomes. Large prospective cohorts of Ebola survivors with HIV are still lacking, and it remains unclear whether HIV-related factors affect persistence duration, transmission risk, tissue compartmentalization, or long-term sequelae. Addressing this gap represents an important priority for Ebola survivorship research. Consequently, any proposed interaction between HIV-associated immune dysfunction and Ebola virus persistence should currently be regarded as a biologically plausible hypothesis rather than an established clinical association.

The objective of raising these hypotheses is not to overstate existing data, but rather to identify a biologically plausible and clinically relevant intersection that has received limited scientific attention. As survivorship increasingly becomes a central component of Ebola medicine, understanding how pre-existing immune dysfunction may shape persistence biology could have implications extending from survivor follow-up strategies to outbreak preparedness and transmission prevention policies.

Beyond HIV-associated immune dysfunction, biological sex may represent another host determinant contributing to the marked heterogeneity observed among Ebola survivors with respect to viral clearance and long-term persistence. Sex-related differences in antiviral immunity have been described across multiple viral infections and are largely driven by genetic and immunological mechanisms linked to the X chromosome. These differences influence both the magnitude of innate immune activation and the efficiency of antiviral responses, suggesting that biological sex may contribute to interindividual variability in viral clearance and long-term reservoir maintenance.

Among the best-characterized X-linked mediators, Toll-like receptor 7 (TLR7) plays a pivotal role in the recognition of viral single-stranded RNA and the induction of type I interferon responses. Experimental evidence has shown that intact TLR7 signaling is required for the development of effective adaptive immune responses capable of preventing persistent viral infection, highlighting the importance of this pathway in long-term antiviral control [56]. More recently, the X-linked host factor DDX3X has emerged as another potential regulator of sex-specific antiviral immunity. DDX3X has been shown to enhance interferon-α production by plasmacytoid dendritic cells following TLR activation and may contribute to the stronger innate antiviral responses frequently observed in females [57].

Although these mechanisms have not yet been specifically investigated in Ebola survivors, they provide a biologically plausible framework through which host genetic background and sex-dependent immune responses could influence viral clearance, persistence dynamics, and the maintenance of tissue reservoirs. Whether these pathways modify Ebola virus persistence differently in people living with HIV remains completely unexplored. However, the coexistence of HIV-associated immune dysregulation and X-linked differences in antiviral immunity provides a biologically plausible framework that deserves further investigation.

These observations should not be interpreted as evidence that biological sex directly determines Ebola virus persistence. Rather, they identify additional host-related variables that may interact with chronic immune activation, tissue-specific immune regulation, and HIV-associated immune dysfunction. Future studies integrating HIV status, biological sex, and detailed immune profiling may help explain why only a subset of survivors develops prolonged viral persistence despite apparently similar clinical recovery and may identify novel host determinants of reservoir maintenance.

In this regard, future studies including people living with HIV could help clarify the long-term dynamics of Ebola virus persistence, immune recovery, and reservoir biology. Major unanswered questions regarding Ebola virus persistence and the potential role of HIV-associated immune dysfunction are summarized in Box 1.

Box 1 **Conceptual and clinical challenges in Ebola virus persistence research.**

▪Determinants of persistence heterogeneity
   The reasons why some Ebola survivors rapidly clear viral reservoirs whereas others exhibit prolonged RNA persistence remain poorly understood. Host immune status, severity of acute disease, tissue-specific immune regulation, and underlying comorbidities may all contribute to this variability [10,15,17,18].
▪Replication-competent virus versus residual viral RNA
   One of the major unresolved questions concerns whether persistent RNA detection consistently reflects biologically active virus capable of transmission or recrudescence. Improved molecular and virological approaches are needed to better define the infectious potential of persistent reservoirs [12,13,34,35].
▪HIV-associated immune dysfunction and viral clearance
   Although direct evidence remains limited, chronic immune activation, T-cell exhaustion, and impaired antiviral surveillance observed in people living with HIV could theoretically influence Ebola persistence dynamics and long-term reservoir maintenance [22,23,24,25,26,27,28,29,33].
▪Persistence-associated outbreak resurgence
   Increasing evidence suggests that persistent viral reservoirs may occasionally contribute to delayed transmission events and outbreak re-emergence, challenging traditional paradigms focused exclusively on zoonotic spillover [12,13].
▪Long-term survivor-centered management strategies
   Future preparedness models may require integrated approaches combining survivor monitoring, reproductive counseling, neurological and ophthalmologic follow-up, genomic surveillance, and HIV care integration in high-prevalence settings [16,41,42,47,53].

## 6. Clinical and Public Health Implications

The recognition that Ebola virus may persist long after apparent clinical recovery has important consequences for both survivor management and outbreak preparedness. Persistence is no longer simply a laboratory observation confined to research settings; rather, it represents a clinically relevant phenomenon with implications for counseling, surveillance strategies, reproductive health, and long-term follow-up programs [5,12,16].

Among the various reservoirs identified to date, the male genital tract remains the most operationally relevant from a public health perspective. The prolonged detection of Ebola virus RNA in semen led to the implementation of structured semen testing and counseling programs in several affected countries following the West African outbreak [10,16]. These initiatives aimed not only to reduce the risk of sexual transmission, but also to provide survivors with guidance regarding condom use, sexual practices, fertility concerns, and family planning decisions [16,53].

These programs highlighted the complex balance between transmission prevention and survivor stigma. Many survivors experienced anxiety, social isolation, and uncertainty related to prolonged RNA positivity and fears of infecting partners [33,53]. Public health strategies therefore require careful integration of virological surveillance with psychosocial support and community-based education. Excessively punitive or alarmist approaches risk reinforcing stigma and undermining trust in survivor monitoring programs.

The persistence of Ebola virus within ocular and neurological compartments also carries significant clinical implications. Ophthalmologic complications, including uveitis and chronic inflammatory eye disease, may substantially impair quality of life among survivors [41,42,43]. Similarly, neurological sequelae and potential CNS persistence raise concerns regarding delayed complications and recrudescence phenomena [40,48]. These findings support the need for multidisciplinary long-term follow-up strategies incorporating ophthalmologic, neurological, infectious disease, and mental health expertise.

The possibility that underlying immune dysfunction may influence persistence dynamics further complicates survivor management. Although evidence specifically involving people living with HIV remains limited, chronic immune activation and impaired antiviral responses may theoretically affect viral clearance and inflammatory recovery [22,23,24,25,26,27]. In regions where HIV prevalence remains high, integrated approaches combining HIV care and Ebola survivor follow-up may therefore become increasingly relevant.

Beyond the potential biological interaction between HIV-associated immune dysregulation and Ebola virus persistence, several observations from West Africa also highlight a broader Ebola-HIV interface relevant to survivor care and outbreak preparedness. During and after the 2013–2016 Ebola outbreak, important disruptions in HIV testing, linkage to care, and ART delivery were documented in Liberia and neighboring countries, emphasizing the vulnerability of HIV services during large-scale epidemics [20,58,59]. In parallel, Ebola-related cohorts identified persistent challenges in HIV diagnosis and continuity of care among people living with HIV (PWH). Although these findings do not directly demonstrate an effect of HIV on Ebola virus persistence, they support the importance of systematically incorporating HIV-related variables into future Ebola survivor studies. In addition, studies evaluating Ebola vaccination strategies in PWH showed that individuals with well-controlled HIV infection were able to mount robust immune responses, supporting the feasibility of including PWH in future survivor and preparedness research frameworks [60].

Such integration could offer several advantages. HIV clinics and antiretroviral therapy programs already provide longitudinal monitoring infrastructures that may facilitate follow-up of Ebola survivors living with HIV. Conversely, survivor programs could help identify individuals requiring broader infectious disease assessment, reproductive counseling, or immunological evaluation. At present, however, these intersections remain largely unexplored within current healthcare frameworks.

Another important consideration concerns outbreak preparedness. The recognition that viral persistence may contribute to delayed flare-ups has challenged the traditional assumption that epidemic re-emergence primarily reflects new zoonotic spillover events [12]. Survivor-associated persistence introduces a more complex epidemiological landscape in which long-term reservoirs may occasionally sustain transmission potential years after the apparent end of an outbreak. This observation has implications for surveillance systems, survivor registries, and genomic monitoring strategies.

At the same time, it is essential to avoid overstating the epidemiological role of persistence-related transmission. Available evidence suggests that such events remain relatively uncommon compared with acute outbreak transmission dynamics [13]. Nevertheless, even infrequent transmission events may carry substantial consequences given the high pathogenicity of Ebola virus and the possibility of renewed epidemic spread. Public health policies must therefore balance vigilance with realistic risk communication.

The broader implications of Ebola persistence also extend into the evolving field of post-acute viral syndromes. Increasing recognition of chronic inflammatory sequelae and persistent viral reservoirs across multiple infections has reshaped understanding of recovery after severe viral disease [28,29]. In this context, Ebola survivorship may represent one of the clearest examples of how incomplete viral clearance and immune dysregulation can intersect to generate prolonged clinical and epidemiological consequences.

Future preparedness strategies will likely require a more integrated vision of Ebola control, extending beyond acute outbreak containment toward long-term survivor-centered approaches. Such models may include: prolonged survivor follow-up programs; access to reproductive and sexual health services; integrated HIV and infectious disease care; genomic surveillance of recrudescence events; and improved understanding of persistence biology within immune-privileged compartments.

**Figure 3:**
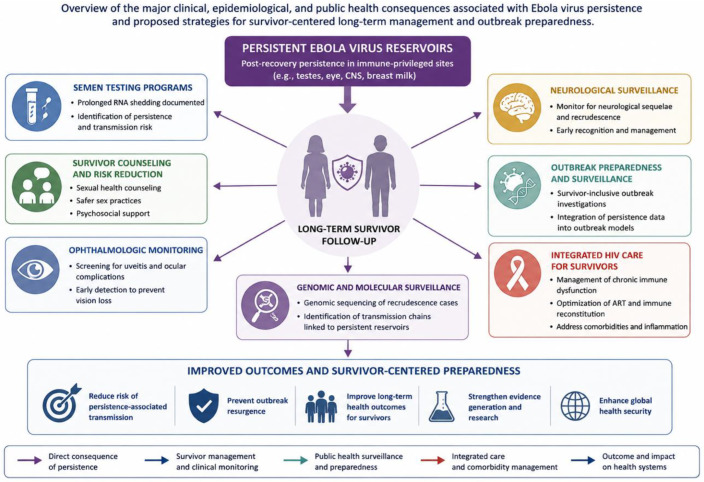
Clinical and public health implications of persistent Ebola virus reservoirs. Overview of the principal clinical, epidemiological, and public health consequences associated with Ebola virus persistence after clinical recovery. The figure summarizes the role of long-term survivor follow-up, semen testing programs, counseling strategies, ophthalmologic and neurological surveillance, genomic monitoring of recrudescence events, outbreak preparedness, and integrated HIV care as components of survivor-centered management and long-term preparedness frameworks. Abbreviations: ART, antiretroviral therapy; CNS, central nervous system; RNA, ribonucleic acid.

As survival rates improve through advances in supportive care, vaccines, and therapeutics, the long-term management of survivors may become an increasingly important dimension of Ebola medicine and global health preparedness. The major clinical and public health implications associated with persistent Ebola virus reservoirs and proposed survivor-centered management strategies are summarized in Figure 3.

## 7. Future Directions and Research Priorities

Despite the growing recognition of Ebola virus persistence as a clinically relevant phenomenon, major uncertainties continue to limit current understanding of its biological mechanisms, epidemiological significance, and long-term consequences. Many of the most important questions surrounding persistence remain unanswered, particularly regarding the factors regulating reservoir maintenance and incomplete viral clearance.

One of the principal limitations of existing evidence is the relative scarcity of prospective longitudinal studies integrating virological, immunological, and clinical data in survivor populations. Most available investigations have focused either on isolated reservoirs, such as semen or ocular tissues, or on specific post-Ebola sequelae without fully exploring the interaction between persistence and systemic immune dysfunction [5,17,18,33]. Future studies will likely require more integrated multidisciplinary approaches capable of simultaneously evaluating viral kinetics, immune activation profiles, tissue-specific persistence, and long-term clinical outcomes.

In particular, the role of host immune status in determining persistence dynamics remains poorly defined. While persistent inflammatory activation and immune dysregulation have been documented among Ebola survivors [17,18], the mechanisms linking these abnormalities to reservoir biology remain incompletely understood. Better characterization of T-cell responses, interferon signaling pathways, macrophage activation, and local tissue immunity may help clarify why some individuals experience prolonged persistence whereas others rapidly achieve viral clearance.

Within this context, the role of HIV-associated immune dysregulation in Ebola virus persistence remains largely unexplored. To date, large-scale survivor cohorts stratified by HIV status are lacking, and it remains unclear whether HIV-related immune alterations influence the duration, compartmentalization, or transmissibility of persistent Ebola virus reservoirs. Current evidence is therefore limited to isolated observations and biological plausibility [52]. Prospective HIV-stratified survivor cohorts could help clarify the interplay between chronic immune dysregulation and post-Ebola virus persistence.

Likewise, future survivor cohorts should evaluate whether sex-dependent immune pathways, including X-linked antiviral regulators such as TLR7 and DDX3X, contribute to interindividual differences in viral clearance, persistence, and long-term reservoir maintenance. Incorporating biological sex into immunological and virological studies may help explain the marked heterogeneity observed among survivors and identify additional host determinants of Ebola virus persistence.

Given the scarcity of direct clinical data in Ebola survivors with HIV, and the limited understanding of other host determinants of persistence, future research should move beyond theoretical plausibility and incorporate systematically measurable virological, immunological, and clinical endpoints within HIV-stratified survivor cohorts. Proposed variables that may help clarify the relationship between HIV-associated immune dysregulation and post-acute Ebola virus persistence are summarized in Table 4.

Future research should also address the distinction between residual viral RNA detection and replication-competent virus. Emerging evidence has also raised the possibility that latency-like mechanisms could contribute to Ebola virus reservoir dynamics and long-term persistence, although these hypotheses still require further validation [58]. Although RNA persistence is well documented, the infectious potential of persistent reservoirs likely varies considerably across tissues and individuals [10,11]. Improved molecular and virological techniques capable of distinguishing inactive viral remnants from biologically active virus would substantially refine current understanding of transmission risk and recrudescence potential.

Another important area concerns the relationship between viral persistence and chronic inflammatory disease. Persistent immune activation following Ebola recovery may contribute not only to clinical sequelae, but also to the maintenance of local viral reservoirs themselves. Similar bidirectional interactions between inflammation and viral persistence have been observed in other chronic viral conditions and immunocompromised states [22,27,28]. Clarifying these interactions may ultimately improve strategies aimed at reducing long-term complications among survivors.

The increasing availability of animal models and translational studies may further accelerate progress in this field. Experimental models have already provided important insights regarding testicular persistence, ocular involvement, CNS recrudescence, and tissue-specific viral tropism [6,36,38,39,40,44]. Future studies combining animal models with advanced immunological profiling and single-cell analyses may help identify the cellular niches responsible for reservoir maintenance.

From a public health perspective, additional work is needed to optimize survivor follow-up strategies while minimizing stigma and social harm. The implementation of semen testing and counseling programs represented a major advance following the West African outbreak [16]. However, long-term sustainability, accessibility, and integration with broader survivor care remain ongoing challenges, particularly in resource-limited settings.

Insights derived from other persistent viral infections and immunocompromised states may also help contextualize how impaired immune surveillance can influence viral clearance and long-term inflammatory sequelae. In this regard, the COVID-19 pandemic renewed interest in post-acute viral syndromes and persistence-related host–pathogen interactions, although direct parallels with Ebola virus biology should be interpreted cautiously.

Ultimately, future Ebola research is likely to shift from an exclusive focus on acute outbreak containment toward a more comprehensive understanding of long-term survivorship biology. In this evolving framework, viral persistence, immune dysregulation, and host-specific determinants of clearance may emerge as central themes shaping the next generation of Ebola medicine and preparedness strategies.

## 8. Relevance for the Current Bundibugyo Ebolavirus Outbreak

The ongoing Bundibugyo virus outbreak in the Democratic Republic of the Congo has brought renewed attention to several unresolved questions in ebolavirus biology, particularly those related to viral persistence and long-term survivor outcomes [61]. While most current knowledge derives from outbreaks caused by Zaire ebolavirus, much less is known about the post-acute phase of Bundibugyo virus infection. Whether BDBV can persist in immune-privileged sites such as the male genital tract, the eye, or the central nervous system remains uncertain. Similarly, there is currently insufficient data to assess the potential risk of persistence-associated transmission or late recrudescence following recovery from BDBV infection. Nevertheless, lessons learned from previous Ebola outbreaks suggest that these issues warrant careful investigation as survivor cohorts from the current outbreak are followed over time. The present outbreak may therefore provide a valuable opportunity to determine whether mechanisms of persistence described for EBOV are shared across different ebolavirus species. Prospective survivor studies could help clarify the interplay between host immune responses, tissue-specific viral persistence, and long-term clinical sequelae. Given the substantial overlap between Ebola-endemic regions and areas with a high burden of HIV infection, such studies may also offer insights into whether HIV-associated immune dysfunction affects post-acute viral clearance or the maintenance of viral reservoirs after BDBV infection. Although these questions remain largely unanswered, integrating survivor follow-up and persistence-focused research into outbreak response activities could generate important evidence for both patient management and future preparedness efforts [61].

## 9. Conclusions

The understanding of Ebola virus disease has evolved substantially over the past decade. Once considered primarily an acute and self-limited infection, EVD is now increasingly recognized as a condition capable of generating prolonged viral persistence, chronic immune dysregulation, and delayed clinical or epidemiological consequences.

Persistent Ebola virus reservoirs or viral material have been documented in multiple immune-privileged sites, including the male genital tract, ocular tissues, the central nervous system, and breast milk. Importantly, persistence is not merely a laboratory curiosity. Molecular evidence of sexual transmission and outbreak resurgence linked to survivor-associated reservoirs has demonstrated that persistent viral RNA and, in selected cases, replication-competent virus may retain clinical and public health relevance. Nevertheless, the biological significance of prolonged viral RNA detection remains incompletely understood, as molecular positivity does not necessarily indicate the presence of viable infectious virus. Distinguishing residual viral RNA from replication-competent virus remains one of the major challenges in defining the true clinical significance of Ebola virus persistence.

At the same time, increasing evidence suggests that Ebola survivors frequently exhibit sustained inflammatory activation and prolonged immune dysfunction. These findings support a more complex model of post-Ebola biology in which viral persistence and host immune responses may interact dynamically over extended periods.

Within this context, the contribution of HIV-associated immune dysregulation remains insufficiently explored. Although available evidence does not establish a definitive association between HIV infection and prolonged Ebola virus persistence, current knowledge remains largely indirect and is primarily supported by biological plausibility rather than direct clinical observations. Chronic immune activation, T-cell exhaustion, altered interferon signaling, and impaired antiviral surveillance in people living with HIV may, in theory, influence viral clearance dynamics and reservoir maintenance.

This review does not aim to overstate the existing evidence. Rather, it seeks to highlight a neglected intersection between Ebola survivorship and chronic immune dysregulation that may deserve greater scientific attention, particularly in regions where both conditions are prevalent. Understanding how host immune status influences persistence biology could have implications extending from survivor monitoring and counseling to outbreak preparedness and transmission prevention strategies.

As survival rates improve and survivor populations continue to grow, the long-term management of EVD will likely become an increasingly important component of global health preparedness. In this emerging landscape, viral persistence should no longer be viewed as an isolated post-acute phenomenon, but rather as part of a broader framework involving reservoir biology, immune regulation, and chronic host–pathogen interactions.

Further multidisciplinary research integrating virology, immunology, clinical medicine, and public health will be essential to clarify the true significance of Ebola virus persistence and its potential interaction with HIV-associated immune dysfunction. Future studies should integrate standardized molecular assays, virological confirmation of replication-competent virus whenever feasible, longitudinal immune profiling, and standardized clinical follow-up to better distinguish established evidence from biologically plausible hypotheses and clarify the mechanisms governing long-term viral persistence.

## Figures and Tables

**Table 1:** Current levels of evidence regarding Ebola virus persistence and the proposed role of HIV-associated immune dysregulation.

Evidence Domain	Strength of Evidence	Main Interpretation	Key References
Ebola RNA persistence in semen	Strong	Documented in longitudinal survivor cohorts with prolonged RNA detection after clinical recovery	[10,11,14,15]
Sexual transmission from survivors	Strong/moderate	Genomic evidence supports rare but real post-recovery transmission events	[12,13,30]
Survivor-associated outbreak resurgence	Strong for selected events	Persistent reservoirs may contribute to delayed outbreak re-emergence	[12]
Post-Ebola immune dysfunction	Moderate/emerging	Survivors may exhibit prolonged inflammatory and immune abnormalities	[17,18,31,32]
Ebola persistence in people living with HIV (PWH)	Very limited	Evidence currently limited to isolated observations, with no prospective studies demonstrating an association with prolonged Ebola virus persistence.	[33]
HIV-associated immune dysregulation as a potential modifier	Hypothesis-generating	Supported by indirect immunological evidence but not yet demonstrated in clinical Ebola survivor cohorts.	[22,23,24,25,26,27,28,29,34,35]

Summary of the current levels of evidence supporting Ebola virus persistence, persistence-associated transmission, post-Ebola immune dysfunction, and the proposed interaction between HIV-associated immune dysregulation and post-acute Ebola reservoir biology. Abbreviations: HIV, human immunodeficiency virus; PWH, people living with HIV; RNA, ribonucleic acid.

**Table 2:** Key evidence supporting persistence-associated transmission and outbreak resurgence in Ebola virus disease.

Finding	Main Evidence	Clinical/Public Health Implication	Key References
Prolonged semen persistence	Longitudinal survivor cohorts documenting persistent Ebola virus RNA in semen for months or years	Need for prolonged survivor follow-up and semen testing programs	[10,11,14,15]
Sexual transmission from survivors	Molecularly linked transmission events from persistent reservoirs	Persistence-associated transmission risk	[13,32,35]
Survivor-associated outbreak resurgence	Genomic evidence linking outbreak recurrence to persistent infection	Revision of traditional outbreak paradigms	[12]
Neurological recrudescence	CNS persistence and delayed disease reactivation	Long-term neurological surveillance needs	[40,47]
Ebola RNA persistence in a survivor living with HIV	Ebola RNA persistence reported in PWH survivor	Supports biological plausibility but does not establish a causal association between HIV-associated immune dysfunction and prolonged Ebola virus persistence.	[52]

Summary of the main clinical, molecular, and epidemiological findings supporting the role of persistent Ebola virus reservoirs in sexual transmission, recrudescence phenomena, and outbreak re-emergence. Abbreviations: CNS, central nervous system; RNA, ribonucleic acid; PWH, People living with HIV.

**Table 3:** Mechanisms of HIV-associated immune dysfunction potentially relevant to Ebola virus persistence.

HIV-Associated Mechanism	Potential Relevance to Ebola Persistence	Key References
Chronic immune activation	Persistent inflammatory environment that may impair effective viral clearance	[22,23,27]
T-cell exhaustion	Potential reduction in antiviral cellular responses and impaired elimination of infected cells	[23,24,25,26,27]
Persistent interferon signaling	Potential alteration of CD8^+^ T-cell metabolic function and antiviral activity	[24]
Immune checkpoint activation	Potential maintenance of dysfunctional T-cell phenotypes and incomplete immune surveillance	[25,26]
Monocyte/macrophage dysfunction	Potential contribution to viral reservoir maintenance and inflammatory signaling	[22,27,54]
Chronic inflammatory dysregulation	Possible amplification of post-Ebola inflammatory sequelae	[17,18,22,27]
Viral reservoir biology	Conceptual parallels with sanctuary-site persistence and incomplete viral eradication	[37,55]

Overview of the principal immunological alterations described in people living with HIV that could theoretically influence Ebola virus clearance, viral persistence, or long-term reservoir maintenance. The proposed mechanisms are based on established knowledge of HIV immunopathogenesis and should be interpreted as biologically plausible pathways rather than direct evidence of Ebola virus persistence. Abbreviations: CD8, cluster of differentiation 8; HIV, human immunodeficiency virus; PWH, People living with HIV.

**Table 4:** Proposed endpoints for future HIV-stratified Ebola survivor cohorts.

Endpoint Category	Suggested Variables/Endpoints
Virological endpoints	Time to semen RT-PCR negativity; intermittent positivity after apparent clearance; Ct values; compartment-specific RNA detection; sequencing; viral culture where feasible.
HIV-related endpoints	HIV status; CD4+ T-cell count; nadir CD4+ T-cell count; CD4/CD8 ratio; HIV viral load; ART exposure; time on ART; history of treatment interruption.
Immune endpoints	HLA-DR/CD38 expression; PD-1, TIM-3, LAG-3; IFN-stimulated gene signatures; IL-6; CRP; sCD14; sCD163; monocyte/macrophage activation markers; TLR7- and DDX3X-related immune signatures; sex-stratified immune profiling.
Clinical endpoints	Ocular disease; neurological sequelae; reproductive counseling needs; sexual transmission risk; psychosocial burden; long-term inflammatory symptoms.

Proposed virological, immunological, HIV-related, and clinical variables that could be systematically evaluated in future HIV-stratified Ebola survivor cohorts to better characterize the relationship between immune dysregulation and post-acute Ebola virus persistence. Abbreviations: ART, antiretroviral therapy; CRP, C-reactive protein; HIV, human immunodeficiency virus; IFN, interferon; IL-6, interleukin-6; RT-PCR, reverse transcription polymerase chain reaction.

## Data Availability

No datasets were generated or analyzed during the current study. All information discussed is derived from previously published sources cited in the manuscript.
